# Elevated Plasma Concentrations of Vitamin D-Binding Protein Are Associated with Lower High-Density Lipoprotein and Higher Fat Mass Index in Overweight and Obese Women

**DOI:** 10.3390/nu13093223

**Published:** 2021-09-16

**Authors:** Leila Setayesh, Abbas Amini, Reza Bagheri, Nariman Moradi, Habib Yarizadeh, Omid Asbaghi, Krista Casazza, Mir Saeed Yekaninejad, Alexei Wong, Katsuhiko Suzuki, Khadijeh Mirzaei

**Affiliations:** 1 Department of Community Nutrition, School of Nutritional Sciences and Dietetics, Tehran University of Medical Sciences, Tehran 1417613151, Iran; setayesh.leila@yahoo.com (L.S.); habib.yari72@yahoo.com (H.Y.); 2 Department of Mechanical Engineering, Australian College of Kuwait, Safat 13015, Kuwait; abbasust@gmail.com; 3 Department of Exercise Physiology, University of Isfahan, Isfahan 8174673441, Iran; will.fivb@yahoo.com; 4 Cellular and Molecular Research Center, Research Institute for Health Development, Kurdistan University of Medical Sciences, Sanandaj 6617713446, Iran; nariman8463@yahoo.com; 5 Cancer Research Center, Shahid Beheshti University of Medical Sciences, Tehran 1416753955, Iran; omid.asbaghi@gmail.com; 6 Marieb College of Health and Human Services, Florida Gulf Coast University, Fort Myers, FL 33965, USA; krista.casazza@gmail.com; 7 Department of Epidemiology and Biostatistics, School of Public Health, Tehran University of Medical Sciences, Tehran 1417613151, Iran; yekaninejad@yahoo.com; 8 Department of Health and Human Performance, Marymount University, Arlington, VA 22207, USA; awong@marymount.edu; 9 Faculty of Sport Sciences, Waseda University, Tokyo 2-579-15, Japan

**Keywords:** vitamin D binding protein, vitamin D, 25(OH)D, obesity, cardiovascular risk factors, lipid profile, adiposity marker, blood pressure

## Abstract

(1) **Background:** Observational studies have established that vitamin D-binding protein (DBP) and 25-hydroxyvitamin D_3_ (25(OH)D) concentrations are the major factors affecting the bioavailability of 25(OH)D. It has also been shown that poor 25(OH)D bioavailability elevates the risk of obesity and its related cardio-metabolic disorders. However, the relationship between 25(OH)D and DBP concentrations with cardio-metabolic risk factors in overweight and obese cohorts has not been established. Consequently, we evaluated the association between DBP and 25(OH)D concentrations with lipid profile, blood pressure (BP), and body composition in overweight and obese women. (2) **Methods:** In this cross-sectional study of 236 overweight and obese women, DBP and 25(OH)D concentrations were measured using an enzyme-linked immunosorbent assay. Body composition was assessed via bioelectrical impedance analysis. Lipid profile and BP were assessed by an auto-analyzer and digital BP monitor, respectively. The associations were examined by multivariate logistic regression. (3) **Results**: The indicated showed an inverse relationship between DBP and high-density lipoprotein (HDL) (*p* = 0.010) concentrations (where individuals with higher DBP had lower HDL) which, after adjusting for possible cofounders, remained significant (*p* = 0.006). Moreover, DBP concentration was positively associated with fat mass index (FMI) after adjustment (*p* = 0.022). No significant relationships were observed among 25(OH)D and target variables. (4) **Conclusions**: In conclusion, lower concentrations of HDL and higher values of FMI are associated with higher concentrations of DBP in overweight and obese women. These findings present novel awareness regarding the association of DBP with some metabolic and body composition variables in overweight and obese women. However, a two-way causal relationship between DBP and target variables should be considered.

## 1. Introduction

Prior research indicates that inadequate vitamin D concentrations may exacerbate the pathology of various chronic diseases, including type 2 diabetes mellitus, abdominal obesity, and dyslipidemia [1,2,3]. Beyond the physiological interactions, lifestyle factors including smoking, physical inactivity, poor diet, geographic location, and racial/ethnic differences influence 25-hydroxyvitamin D_3_ (25(OH)D) status [1,4,5]. Moreover, the independent and interactive effects of numerous factors, including body mass index (BMI) and body fatness, affect variations in vitamin D binding protein (DBP) [6].

Circulating 25(OH)D, whether ingested or synthesized in the skin, is highly dependent upon DBP. A significant proportion of 25(OH)D (~88% of 25(OH)D and ~85% of biologically active 1.25(OH)D) are bound to DBP, with the remainder bound to albumin (10–13%) and a free fraction (0.1–2%) [7]). It has been proposed that DBP may be an essential factor responsible for determining free and bioavailable 25(OH)D concentrations [8]. This challenges the “free hormone” hypothesis, where only unbounded molecules migrate into cell membranes and have particular metabolic effects [9]. Furthermore, free and bioavailable vitamin D have been positively correlated with 25(OH)D and inversely with DBP concentrations [10,11].

Despite the effect of DBP concentrations on 25(OH)D, several studies have shown an independent association of DBP with the risk of several cardio-metabolic disorders, such as ischemic stroke and vascular inflammation [12,13,14]. A vast majority of clinical trials have focused on assessing the total of 25(OH)D concentrations [14] without considering DBP concentrations. Indeed, DBP as a determinant parameter for the 25(OH)D concentrations and an independent risk factor for metabolic diseases has not been well-distinguished, especially in communities with a high rate of chronic diseases and genomic heterogeneity, i.e., the Middle East. In addition, there are not enough systematic clinical evaluations of DBP about the chronic risk factors among overweight and obese women in this region. Therefore, our investigation aimed to evaluate the relationship between 25(OH)D and DBP concentrations with cardio-metabolic risk factors. As an independent factor and 25(OH)D concentrations, we hypothesized that DBP might relate to lipid profile, blood pressure (BP), anthropometric, and body composition in overweight and obese Iranian women.

## 2. Materials and Methods

### 2.1. Population Study

A cohort of 236 healthy overweight and obese women (BMI > 25, 18–56 years of age) in the follicular phase with moderate exercise from Tehran, Iran (latitude: 32°25′14.67″ N; elevation: 1189 m), participated in this cross-sectional study. Exclusion criteria were age <18 or >60 years, pregnancy or lactation, alcohol consumption, drug abuse, smoking, and recent changes in dietary habits. Participants were also excluded if they had acute or chronic inflammatory diseases and a history of hypertension, cardiovascular disease (CVD), diabetes mellitus, impaired renal and liver function, thyroid disease, and other malignancies. Women reporting involuntary or voluntary body mass fluctuation over the past year, regular use of medications (including oral contraceptives, antibiotics, osteoporosis therapies, anti-inflammatory drugs, statins, and other hypolipidemic agents) or nutritional supplements (including antioxidants, probiotics, vitamins such as D, and minerals such as calcium) within two months before the enrollment for this study were also excluded. In conjunction with Tehran University of Medical Sciences, the protocol was approved by the Ethics Committee of Tehran University of Medical Sciences (IR.TUMS.VCR.REC.1397.979). All the procedures were performed in accordance with the ethical standards of the responsible committee on human experimentation (institutional and national) and with the Declaration of Helsinki 1975, as revised in 2013 [15]. All participants signed informed consent forms and completed a self-reported health status questionnaire prior to the initiation of the study.

### 2.2. Study Design

Two visits were conducted: on the first visit, health, dietary, physical activity, and sociodemographic characteristics information was documented, and potential participants were included or excluded from the study from 25 health centers in Tehran. After inclusion, participants were scheduled for their second visit. Data collection occurred at Tehran University of Medical Sciences’ laboratory, where blood sample collection, body composition, and BP measurements were performed between 8–9 a.m. after 12 h of overnight fasting. Participants were enquired to avoid caffeine and alcohol consumption for 24 h and any unusual physical activity for 72 h before the third visit. The time between visits was 24 h.

### 2.3. Anthropometric and Body Composition Measurements

Height was measured via a wall-mounted stadiometer (Seca 711, Hamburg, Germany) and rounded to the nearest = 0.5 cm, while body mass was determined using a calibrated balance beam scale (Seca 711, Hamburg, Germany) and rounded to the nearest = 0.1 kg, respectively. BMI was classified based on the standard of World Health Organization (WHO), where BMI of normal body mass is defined as 18.5–24.9 kg/m^2^; overweight 25.0–29.9; grade I obesity 30.0–34.9; grade II obesity 35.0–39.9; and grade III obesity ≥40.0 [16]. Waist circumference (WC) was measured in cm according to the standard of the National Center for Health Statistics [17].

Bioelectrical impedance analysis (BIA) (InBody 720,Biospace Co., Seoul, Korea) was used to measure total body water (TBW), fat-free mass (FFM), body cell mass (BCM), fat mass (kg), body fat percentage (BFP), and visceral fat area (VFA). The following equation was used to estimate free fat mass index (FFMI) for each participant [18]: *FFMI* (kg/m^2^) = *fat free mass* (kg)/(height(m))^2^. Moreover, fat mass index (FMI) [19] was calculated via these equations: *FMI* (kg/m^2^) = *fat mass* (kg)/(height(m))^2^.

The same operator identified the measurements (a nutritionist experienced in body composition measurements and nutritional assessments). Moreover, participants were required to void their bladder before measurements.

### 2.4. BP Assessment

Using standard procedures, BP was measured in mmHg using a digital BP monitor (HEM 7120, Omron, Kyoto, Japan) after sitting quietly for at least 5 min. Two measurements at 1 min intervals were collected and averaged.

### 2.5. Biochemical Assessment

Fasting blood samples were collected from the antecubital vein using standard procedures. The serum was centrifuged, liquated, and stored at a temperature of −80 °C for further analyses. All biochemical analyses, including total cholesterol (TC) and triglycerides (TG) were performed using a Roche Modular Analytics System in our laboratory. Low-density lipoprotein (LDL) and high-density lipoprotein (HDL) were determined using a homogeneous enzymatic assay for a direct quantitative determination. The serum concentration of 25(OH)D was measured using an ELIZA kit (IDS kit, Bolden, United Kingdom). The vitamin deficiency was defined at a serum concentration of vitamin D < 20 ng/mL (<50 nmoL/L), insufficiency in the range of 21–29 ng/mL (52.5–72.5 nmoL/L), and normal ≥ 30 ng/mL (≥75 nmoL/L) [20]. DBP (monoclonal R&D system kit, Boston, USA) and 25(OH)D concentrations were measured using an enzyme-linked immunosorbent assay (ELISA). The concentration of free 25(OH)D was calculated using the following formula [7]. The albumin concentration was estimated at 40 mg/dL and the concentration of free 25(OH)D in pmol/L [21].

(1) $$\mathrm{Calculated}\;\mathrm{free}\;25\left(\mathrm{OH}\right)D=(\mathrm{Total}\;25(\mathrm{OH})D)/(1+(6\times 10^{3}\times (\mathrm{Albumin}))+(7\times 10^{8}\times (\mathrm{DBP})))$$

### 2.6. Dietary Assessment

A 147-item semi-quantitative food frequency questionnaire (FFQ) adjusted for Iranian foods, following Willett [22], was applied to assess the average dietary intake over the past year. Standard methods were used to transform portion sizes of the consumed foods into grams, and a predictable average daily intake of food parameters (including macro and micronutrients) was computed from the FFQ using Nutritionist 4 (Hearst Corporation, San Bruno, CA, USA) food analyzer.

### 2.7. Statistical Analyses

Descriptive statistics of study covariates and outcomes were performed. Visual inspection was utilized for normality, and the extreme data outliers were excluded. The Kolmogorov–Smirnov test evaluated data distribution, and the results were expressed in mean ± standard deviation (SD). Independent sample t-tests were applied to assess relevant variables between two categories of 25(OH)D (cut point: 30) and DBP (cut point: median). A multivariate logistic regression model was used to assess the association of lipid profile, BP, and body composition (coded as 0 for normal and 1 for disorder state) with DBP and 25(OH)D concentrations (response and continuous variables). Results were then adjusted using a multivariate model that included sociodemographic characteristics and reported with odds ratios (OR) and confidence intervals (CI). Statistical significance was set at *p* < 0.05. No mathematical correction was applied for testing multiple associations. Instead, all results, including 95% CIs, and *p*-values are reported. All statistical analyses were performed using SPSS^®^ 21 (SPSS Inc., Chicago, IL, USA).

## 3. Results

### 3.1. Participant’s Characteristics

Participants were on average 36 ± 8 years and had a mean BMI of 31.04 ± 4.31 kg/m^2^. Moreover, mean 25(OH)D, free25(OH)D and DBP were 67.18 ± 3.08 ng/mL, 22.62 ± 16.81 pmol/L, and 435.73 ± 77.50 ng/mL, respectively. This indicated an adequate concentration of 25(OH)D (25(OH)D ≥ 30 ng/mL) in 69% of our participants, while 15.9% had an insufficient concentration (21–29 ng/mL) and 14.2% had 25(OH)D deficiency (<20 ng/mL) (Table 1).

### 3.2. Study Population Characteristics Based on Two Categories of 25(OH)D and DBP Concentrations

The population’s anthropometric characteristics, lipid profile, and BP are presented in Table 2, classified by sufficient or insufficient, i.e., 25(OH)D by < or ≥30 ng/mL. This classification revealed an inverse correlation between 25(OH)D and LDL and fat-free mass index (FFMI), wherein individuals with a lower concentration of 25(OH)D (<30 ng/mL) had a higher concentration of LDL (*p* = 0.044) and higher FFMI (*p* = 0.026). Moreover, a positive relationship between 25(OH)D and free 25(OH)D concentrations was detected. Participants with a higher concentration of 25(OH)D (<30 ng/mL) had a greater concentration of free 25(OH)D (*p* < 0.001).

Table 2 also presents the varied concentration of DBP for the median category (439.28 ng/mL). A positive relationship was observed between BMI and DBP concentration (*p* = 0.049). In addition, a significant inverse relationship was observed between DBP and free 25(OH)D concentration, where individuals with higher than the median DBP concentration had a lower concentration of free 25(OH)D (*p* < 0.001). No meaningful relationship was observed in other variables across the two groups.

### 3.3. Correlation between 25(OH)D and BP, Anthropometric and Lipid Profile

The relationships between 25(OH)D concentration and BP, anthropometric variables, and lipid profile are presented in Table 3. A significant relationship was established between 25(OH)D and free 25(OH)D concentrations in both models (OR = 1.53, CI = 1.13 to 2.08, *p* = 0.006). No other significant association was observed among studied variables and 25(OH)D concentration.

### 3.4. Correlations between DBP and BP, Anthropometric and Lipid Profile

Table 4 shows the association between DBP concentration with free 25(OH)D, BP, anthropometric, and lipid profile. A significant inverse relationship was observed between DBP and HDL concentrations in the crude model (OR = 0.66, CI = 0.48 to 0.90, *p* = 0.010), such that women with higher DBP concentrations had lower HDL concentrations. This inverse association was not altered when potential confounders, including age (years), BMI, and standardized energy intake (kcal) (OR = 0.62, CI = 0.45 to 0.85, *p* = 0.006) were added. In model 2, after adjusting the confounders, the resultant DBP concentration positively correlated with FMI (OR = 1.88, CI = 1.09 to 3.23, *p* = 0.022), where the participants with a greater concentration of DBP significantly increased the risk of FMI.

## 4. Discussion

The objective of this investigation was to explore the relationships between DBP and 25(OH)D with BP, body composition, and lipid profile in overweight and obese women. Briefly, we observed that DBP concentration might be positively associated with FMI and negatively related to HDL. No such relationship was observed for either DBP or 25(OH)D concentrations with other variables. Although most of the participants had adequate 25(OH)D concentrations, the association of 25(OH)D with risk factors for chronic disease raised questions regarding the bioavailability or independent role of DBP.

Leong et al. reported that DBP had a strong observational and causal association with 25(OH)D concentrations [23]. In addition, Bikle et al. [7] reported that the free and total 25(OH)D concentrations could be influenced by variations in DBP concentration. Among the different phenotypes of DBP, the total 25(OH)D can be different from the exact amount free 25(OH)D. Because DBP concentrations vary with factors such as obesity and gender, the measurements of free 25(OH)D and DBP can be considered influential components in several obesity-related disorders [24]. Therefore, evaluating DBP among diverse populations may provide a more comprehensive explanation for the inconsistent results. This is in line with the assertion that excess adipose tissue hinders vitamin D transport and adversely affects vitamin D metabolism [25]. While no studies have explored DBP in overweight and obese Middle Eastern women, some have assessed the relationship of DBP with BMI in other populations and reported conflicting results [26,27,28,29]. We observed a positive association between DBP and FMI; however, no relationship was observed with other body composition variables. Most of our target population (~69%) had > 30 ng/mL concentration of 25(OH)D. As a result, we may conclude that this carrier is associated with FMI (in relation to the bioavailability of 25(OH)D or independent role of DBP), while the mutual association between FMI and DBP cannot be ruled out. By this means, higher FMI may be the cause of higher DBP among overweight and obese women.

Other studies have reported contradicting results, where body mass and BMI had positive [27] or negative [8] associations with DBP, or even no relationship at all [29,30]. Winters et al. suggested that DBP concentrations in 88 women (different race types) had no significant relationship with obesity and 25(OH)D concentrations [29]. Moreover, Naderpoor et al. showed that women with polycystic ovary syndrome had a lower DBP concentration than a healthy control group, with no association between DBP and BMI [31]. Almesri et al. reported that polymorphisms of DBP in the GC (rs2282679, rs4588, rs7041) and VDR (rs12721377) genes were independently associated with BMI and 25OHD3 concentrations, with an evident sex dimorphism [32]. Moreover, Speeckaert et al. observed that the 25(OH)–vitamin D3/DBP ratio negatively correlated with BMI [33]. These outcomes are somewhat in line with our findings. While there was no association between DBP and BMI, FMI, and WHR may serve as better indicators of abdominal/visceral obesity than BMI. The current outcomes may have important clinical implications on the essential physiological role of DBP in obesity and related disorders.

On the other hand, several clinical trials have found that free and bioavailable vitamin D may positively correlate with the 25(OH)D form and inversely with DBP concentrations [10,11]. One possible mechanism is that at higher concentrations of DBP, a lower free fraction of 25(OH)D is transmitted to adipocytes, thus decreasing adipogenesis. When there is a lower concentration of 25(OH)D, the parathyroid hormone (PTH) is elevated and increases cytosolic calcium [34]. This phenomenon inhibits catecholamine-induced lipolysis [35] and promotes fatty acid synthase expression [36], resulting in excess fat accumulation.

We also observed that a significant relationship between DBP and HDL existed even after adjusting the covariates. Individuals with higher DBP had a lower concentration of HDL (less than normal values). The association of DBP with lipoproteins has been previously demonstrated, in which individuals with acromegaly with a higher DBP had higher concentrations of TG and LDL [37]. They also observed higher DBP and lower 25 free (OH) D concentrations while the total 25(OH) D had no substantial changes [37]. Several studies have also illustrated a relationship between 25(OH)D concentration with lipid profiles. Indeed, insufficient 25(OH)D has been linked to unfavorable lipid profile concentrations [38,39]. Moreover, it has been previously observed that vitamin D by itself may be related to lower HDL concentrations [40]. Nevertheless, other studies have reported no relationship between vitamin D and lipid profiles [39,41,42]. Nevertheless, we should bear in mind that adiposity is recognized to greatly impact metabolic parameters and lipids. Obesity is also very frequently associated with low 25(OH)D concentrations [43]. Therefore, it is not surprising that 25(OH)D concentrations would be associated with lipid status, but it is even more likely mediated by obesity. In addition, several studies showed the independent role of DBP in lipid profile alterations. This carrier may be associated with health status, other than its related function to 25(OH)D. Similar to our results, Speeckaert et al. [33] and Naderpoor et al. [31] indicated a negative correlation between DBP and HDL concentrations among Belgians and Australians, respectively.

Recent studies showed that DBP synthesis and its concentrations would increase following a high-fat diet [44]. It was hypothesized that increased fatty acids caused by a high-fat diet accelerated 25(OH)D metabolism to maintain energy balance under a high-energy diet [44]. As such, a possible relationship between DBP and bioavailable 25(OH)D may have an independent role in supporting the healthy concentration of HDL among obese women based on genetic predisposition. Moreover, DBP (alongside the transport of 25(OH)D) as a plasma glycoprotein, has several substantial roles such as modulation of immune and inflammatory responses, bone improvement, transferring of fatty acid, and sequestration of actin that can have independent roles in CVD and thrombosis [45]. Lutsey et al. showed that individuals genetically predisposed to a greater concentration of DBP have a higher risk of heart failure [46]. Indeed, the potential for reverse causality is substantial in cross-sectional studies [47].

Over 70 percent of the women in our study had normal BP (120/80), and we found no major relationship between DBP and BP. Similarly, Hirai et al. [48] and Leong et al. [23] found no significant correlation between DBP and BP in Japanese and Canadian cohorts, respectively. However, a large study evaluating the relationship between BP and 25(OH)D concentrations (*n* = 146,581) reported a borderline relationship between DBP and SBP with 25(OH)D concentrations. Further studies evaluating the relationship between DBP and BP in different populations are warranted.

While our study uncovered relationships and areas for further investigation, it was not without limitations. First, given the cross-sectional and observational nature of the study, we were incapable of predicting exact cause-effect influence. Second, we could not calculate the exact amount of free and bioavailable 25(OH)D due to the lack of blood albumin data. Third, estrogen as one of the factors that may affect DBP was not measured.

## 5. Conclusions

In conclusion, a lower concentration of HDL and higher values for FMI is associated with a higher concentration of DBP in overweight and obese women. The current outcome strengthens existing evidence of some clinical relationships for the physiological role of DBP. Further prospective studies and randomized controlled trials are warranted to confirm and elucidate the effect of DBP concentrations and obesity-related disorders.

## Figures and Tables

**Table 1:** Characteristics and anthropometric parameters, vitamin D-binding protein, and vitamin D, of the target population.

Variable	Minimum	Maximum	Mean	SD
Age (year)	18	56	36.49	8.31
Body mass (kg)	59.5	136.6	80.89	12.45
Height (cm)	142	179	161.38	5.9
BMI (kg/m^2^)	24.2	49.6	31.04	4.31
Body fat percentage	15	54.3	41.53	5.48
WHR (m)	0.81	1.08	0.93	0.05
Fat Mass (kg)	19.4	74.2	34.04	8.69
WC (cm)	80.1	136	99.01	10.05
FMI (kg/m^2^)	6.9	26.9	13.15	3.37
FFMI (kg/m^2^)	14.6	147.8	18.37	7.64
TC (mg/dL)	104	344	185.3	35.77
TG (mg/dL)	37	512	118.38	64.64
HDL-C (mg/dL)	18	87	46.58	10.86
LDL-C (mg/dL)	34	156	95.3	24.12
SBP (mmHg)	76	159	111.7	13.66
Diastolic BP (mmHg)	51	111	77.84	9.58
Energy (kcal)	1028.98	4192.72	2613.04	44.07
DBP (ng/mL)	185.34	798.39	435.74	77.51
25(OH)D (ng/mL)	2.9	168.8	66.65	46.73
Free 25(OH)D (pmol/L)	0.87	78.81	22.62	16.81

Abbreviations: BMI, body mass index; WHR, waist-hip ratio; FMI, fat mass index; FFMI, fat-free mass index; WC, waist circumference; FBS, fasting blood sugar; TG, triglyceride; TC, total cholesterol; HDL-C, high-density lipoprotein; LDL-C, low-density lipoprotein; SBP, systolic blood pressure; DBP, diastolic blood pressure; DBP, vitamin D-binding protein; SD, standard deviation; BP, blood pressure.

**Table 2:** Anthropometric characteristics, blood pressure, and lipid profile of the studied population, grouped based on DBP (nmol/L) and 25(OH)D concentration (ng/mL).

Variable	DBP		*p*-Value	25(OH)D		*p*-Value
	Serum ≤ 439.28 ng/mL	Serum > 439.28 ng/mL		Serum < 30 ng/mL, *N* = 74	Serum ≥ 30 ng/mL, *N* = 162	
Age (year)	35.80 (8.01)	36.57 (9.02)	0.110	35.61 (8.17)	36.61 (8.49)	0.509
Body mass (kg)	79.49 (10.99)	82.43 (13.38)	0.093	81.49 (11.86)	81.012 (12.58)	0.381
Height (cm)	161.41 (5.85)	161.86 (5.52)	0.386	162.10 (5.52)	161.51 (5.52)	0.436
BMI (kg/m^2^)	30.49 (3.73)	31.50 (4.83)	**0.049**	31.14 (4.14)	31.02 (4.49)	0.365
Fat mass (kg)	33.11 (7.69)	35.14 (9.46)	0.146	34.60 (8.10)	34.01 (9.04)	0.110
Body fat percentage	41.08 (5.80)	42.06 (5.26)	0.499	47.13 (5.69)	46.87 (5.48)	0.309
WHR (m)	0.93 (0.05)	0.93 (0.05)	0.711	0.94 (0.05)	1.50 (7.19)	0.269
FMI (kg/m^2^)	12.78 (3.02)	13.53 (3.68)	0.179	13.23 (3.07)	13.16 (3.59)	0.098
FFMI (kg/m^2^)	17.76 (1.39)	19.04 (11.54)	0.140	19.69 (15.28)	17.95 (1.49)	**0.026**
WC (cm)	98.00 (9.04)	100.25 (10.70)	0.100	100.26 (9.93)	98.871 (9.94)	0.193
HDL-C (mg/dL)	46.56 (11.61)	45.81 (10.37)	0.080	47.09 (12.00)	46.02 (10.81)	0.353
LDL-C (mg/dL)	94.18 (22.92)	93.50 (24.90)	0.486	94.18 (26.01)	93.150 (22.64)	**0.044**
TG (mg/dL)	118.91 (66.33)	122.46 (74.85)	0.852	114.85 (62.42)	120.89 (75.15)	0.286
TC (mg/dL)	182.82 (35.47)	186.22 (36.41)	0.727	186.60 (35.29)	184.14 (36.52)	0.707
SBP (mmHg)	110.52 (13.96)	112.78 (13.81)	0.973	112.084 (13.92)	111.76 (13.77)	0.596
Diastolic BP (mmHg)	78.00 (10.59)	77.48 (9.03)	0.192	78.07 (10.66)	77.86 (9.29)	0.218
25(OH)D (ng/mL)	70.10 (47.34)	64.69 (47.49)	0.725	-	-	-
DBP (ng/mL)	-	-	-	441.49 (72.19)	436.76 (80.63)	0.750
free25(OH)D (pmol/L)	26.88 (18.78)	18.78 (13.60)	<0.001	8.60 (3.98)	36.40 (12.70)	<0.001

Abbreviations: Data presents the varied levels of DBP for the median category (≤439.28 nmol/L and >439.28 nmol/L) and the basis of 25(OH)D, < or ≥30 ng/mL. BMI, body mass index; WHR, waist-hip ratio; FMI, fat mass index; FFMI, fat-free mass index; WC, waist circumference; FBS, fasting blood sugar; TG, triglyceride; TC, total cholesterol; HDL-C, high-density lipoprotein; LDL-C, low-density lipoprotein; SBP, systolic blood pressure; DBP, diastolic blood pressure; DBP, vitamin D-binding protein. Results were expressed with mean ± standard deviation (SD). A *p*-value in bold denotes a significant difference (*p* < 0.05).

**Table 3:** Correlation between anthropometric measurements, lipid profile, and blood pressure with 25(OH)D.

Variable	Model 1 *			Model 2 ^†^		
	OR	CI	*p*-Value	OR	CI	*p*-Value
BMI (kg/m^2^) ^‡^	0.91	0.70 to 1.91	0.521	0.92	0.70 to 1.21	0.561 ^‡^
WHR (cm) ^‡^	0.92	0.64 to 1.34	0.626	1.22	0.95 to 2.79	0.549 ^‡^
FMI (kg/m^2^) ^‡^	0.88	0.68 to 1.14	0.357	0.88	0.67 to 1.16	0.379 ^‡^
FFMI (kg/m^2^) ^‡^	1.41	0.84 to 1.41	0.510	1.12	0.85 to 1.48	0.172 ^‡^
WC (cm) ^‡^	0.92	0.64 to 1.34	0.697	0.88	0.60 to 1.29	0.529 ^‡^
HDL-C (mg/dL)	0.96	0.70 to 1.32	0.817	0.89	0.63 to 1.26	0.529
LDL-C (mg/dL)	1.20	0.76 to 1.91	0.418	1.33	0.82 to 2.17	0.245
TG (mg/dL)	0.97	0.68 to 1.38	0.895	1.14	0.77 to 1.68	0.493
TC (mg/dL)	0.86	0.64 to 1.17	0.355	0.88	0.16 to 1.21	0.450
SBP (mmHg)	1.12	0.81 to1.55	0.466	1.24	0.83 to 1.76	0.229
Diastolic BP (mmHg)	1.10	0.83 to 1.46	0.503	1.11	0.82 to 1.50	0.477
DBP (ng/mL)	1.06	0.80 to 1.39	0.661	0.97	0.66 to 1.42	0.455
Free 25(OH)D (pmol/L)	1.47	1.17 to 1.83	**0.001**	1.53	1.13 to 208	**0.006**

Logistic regression was applied to assess the correlation. * Model 1: Crude model. ^†^ Model 2: Adjusted for age, body mass index, and standardized energy intake (kcal). ^‡^ Those variables were not adjusted for BMI due to collinearity. The obesity markers (BMI, WC, FFMI, FMI, and WHR) were categorized based on their median into two groups of higher and lower of than the median. BMI, body mass index; WHR, waist-hip ratio; FMI, fat mass index; FFMI, fat-free mass index; WC, waist circumstance; TG, triglyceride; TC, total cholesterol; HDL-C, high-density lipoprotein; LDL-C, low-density lipoprotein; SBP, systolic blood pressure; DBP, diastolic blood pressure; DBP, vitamin D-binding protein; CI, confidence interval; OR, odds ratio. A *p*-value in bold denotes a significant difference (*p* < 0.05).

**Table 4:** Correlations between anthropometric measurements, lipid profile, and blood pressure with DBP.

Variable	Model 1 *			Model 2 ^†^		
	OR	CI	*p*-Value	OR	CI	*p*-Value
BMI (kg/m^2^) ^‡^	1.07	0.83 to 1.36	0.581	1.07	0.83 to 1.38	0.57 ^‡^
WHR^‡^	1.43	0.70 to 2.91	0.322	1.10	0.85 to 1.42	0.451 ^‡^
FMI (kg/m^2^) ^‡^	1.89	0.92 to 1.52	0.172	1.88	1.09 to 3.23	**0.022** ^‡^
FFMI (kg/m^2^) ^‡^	1.08	0.85 to 1.39	0.499	1.10	0.85 to 1.42	0.451 ^‡^
WC (cm) ^‡^	0.83	0.58 to 1.17	0.295	0.83	0.59 to 1.71	0.375 ^‡^
HDL-C (mg/dL)	0.66	0.48 to 0.90	**0.010**	0.62	0.45 to 0.85	**0.006**
LDL-C (mg/dL)	0.99	0.65 to 1.51	0.974	0.91	0.59 to 1.40	0.668
TG (mg/dL)	0.79	0.57 to 1.09	0.159	0.75	0.54 to 1.05	0.092
TC (mg/dL)	1.00	0.76 to 1.33	0.954	0.96	0.72 to 1.28	0.813
SBP (mmHg)	0.94	0.69 to 1.28	0.701	0.89	0.63 to 1.25	0.518
Diastolic BP (mmHg)	1.00	0.76 to 1.31	0.971	0.97	0.73 to 1.28	0.857
25(OH)D (ng/mL)	0.89	0.69 to 1.15	0.382	0.90	0.68 to 1.18	0.857
Free25(OH)D (pmol/L)	0.87	0.66 to 1.15	0.332	0.88	0.66 to 1.17	0.380

Logistic regression was applied to assess the correlation. * Model 1: Crude model. ^†^ Model 2: Adjusted for age (years), body mass index (kg/m^2^), and standardized energy intake (kcal). ^‡^ Those variables were not adjusted for BMI due to its being collinear. The obesity markers (BMI, WC, FFMI, FMI, and WHR) were categorized based on their median into two groups of higher and lower than the median. BMI, body mass index; WHR, waist-hip ratio; FMI, fat mass index; FFMI, fat-free mass index; WC, waist circumference; TG, triglyceride; TC, total cholesterol; HDL-C, high-density lipoprotein; LDL-C, low-density lipoprotein; SBP, systolic blood pressure; Diastolic BP, diastolic blood pressure; DBP, vitamin D-binding protein; VD, 25(OH)D; CI, confidence interval; OR, odds ratio. A *p*-value in bold denotes a significant difference (*p* < 0.05).

## Data Availability

The data presented in this study are available on request from the corresponding author.
